# Supplementary material for: Epstein-Barr virus-driven B cell lymphoma mediated by a direct LMP1-TRAF6 complex
Source: Nat Commun. 2024 Jan 10;15:414. doi: 10.1038/s41467-023-44455-w (PMC10776578; doi:10.1038/s41467-023-44455-w)
Supplement: Supplementary file 1 — Supplementary Information [file 41467_2023_44455_MOESM1_ESM.pdf]

Epstein-Barr virus-driven B cell lymphoma mediated by a direct LMP1-TRAF6 complex

Fabian Giehler, Michael S. Ostertag, Thomas Sommermann, Daniel Weidl, Kai R. Sterz, Helmut Kutz, Andreas Moosmann, Stephan M. Feller, Arie Geerlof, Brigitte Biesinger, Grzegorz M. Popowicz, Johannes Kirchmair, Arnd Kieser

Supplementary Information

Supplementary Figure 1

a

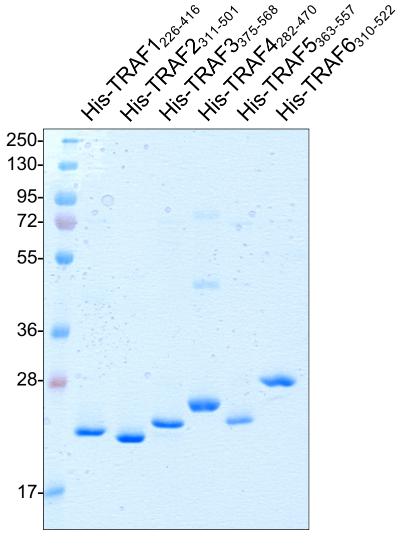

b

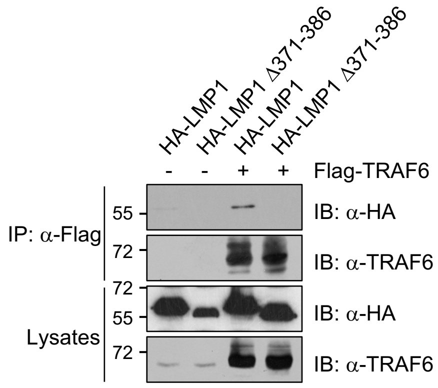

c

#	Protein	AA	Sequence	Mutation	T6	T2
1	CD40	230-259	KQEPQEINFPDDLPGSNTAAPVQETLHGCQ	-	+	+
2	CD40	230-259	KQEPQEINFPDDLPGSNTAAAVAEALHGCQ	A_{250}xAxA	+	-
3	CD40	230-259	KAEAQAINFPDDLPGSNTAAPVQETLHGCQ	A_{231}xAxA	-	+
4	CD40	244-273	GSNTAAPVQETLHGCQPVTQEDGKESRISV	-	-	+
5	CD40	244-273	GSNTAAAVAEALHGCQPVTQEDGKESRISV	A_{250}xAxA	-	-
6	LMP1	198-227	DDSLPHPQQATDDSGHESDSNSNEGRHHLL	-	-	+
7	LMP1	198-227	DDSLPHAQAAADDSGHESDSNSNEGRHHLL	A_{204}xAxA	-	-
8	LMP1	371-386	--------------GDDDDPHGPVQLSYYD	-	+	-
9	LMP1	371-386	--------------GDDDDPHGPVQLSAAD	$A_{384}A_{385}$	-	-
10	LMP1	371-386	--------------GDDDDPHGAVALAYYD	A_{379}xAxA	-	-
11	LMP1	357-386	PHLPTLLLGSSGSGGDDDDPHGPVQLSYYD	-	+	-
12	LMP1	357-386	PHLPTLLLGSSGSGGDDDDPHGAVALAYYD	A_{379}xAxA	-	-

d

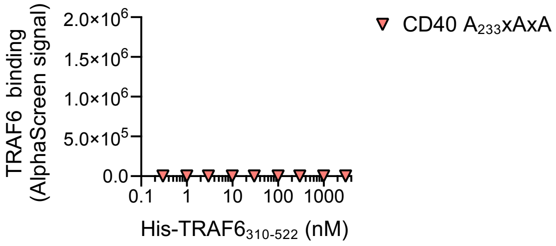

CD40 A_{233}xAxxA A_{233}Q A I N A no binding

e

	-2	-1	0	1	2	3
LMP1 379-384	P	V	Q	L	S	Y
CD40 233-238	P	Q	E	I	N	F
RANK 344-349	P	T	E	D	E	Y
Cellular consensus	P	x	E	x	x	F/Y/D/E
Extended consensus	P	x	E/Q	x	x	F/Y/D/E

f

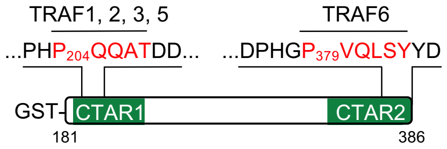

	AA 203-209		AA 378-384	
LMP1 A_{204}xAxA	H A Q A A A D	···	G P V Q L S Y	73.0 ± 9.8 nM
LMP1 Y384D	H P Q Q A T D	···	G P V Q L S D	131.4 ± 28.9 nM

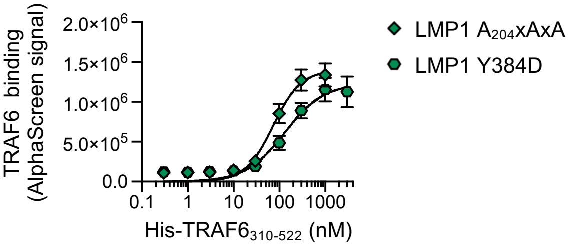

Supplementary Figure 1

Supplementary Figure 1. Additional information and experiments to Figure 1 (direct interaction of LMP1-CTAR2 with TRAF6).

(a) Recombinant His-TRAF preparations. Proteins are visualized by Coomassie staining after SDS-PAGE. Molecular weights are given in kilodaltons (kDa).

(b) Deletion of the sixteen C-terminal amino acids of CTAR2 abolishes the interaction of TRAF6 with LMP1 *in vivo*. HEK293 cells were transfected with Flag-TRAF6 and HA-LMP1 or the HA-LMP1 Δ371-386 mutant. Twenty-four hours post transfection, Flag-TRAF6 was immunoprecipitated via its Flag-tag, and coprecipitating HA-LMP1 was analyzed on immunoblots via an α-HA-tag antibody. One representative result of two independent experiments is shown. Molecular weights are given in kilodaltons (kDa).

(c) Peptide sequences of the arrays shown in Figure 1c. Alanine exchanges are indicated in red. Interaction with TRAF6 or TRAF2 is indicated according to the results shown in Figure 1c.

(d) Absent His-TRAF6$_{310-522}$ interaction with GST-CD40 A$_{233}$xAxxA in AlphaScreen PPI assays. Data are mean values ± SD of three independent experiments. Source data in the Source Data file.

(e) Sequence alignment of the TRAF6 binding sequences of LMP1, CD40, and RANK with the earlier described cellular TRAF6 consensus PxExxF/Y/D/E [46], compared to the extended TRAF6 binding consensus PxE/QxxF/Y/D/E, which emerged from our present study.

(f) His-TRAF6$_{310-522}$ interaction with the GST-LMP1 A$_{204}$xAxA and Y384D mutants. AlphaScreen PPI experiments. K_D values are given. Data are mean values ± SD of four independent experiments. Curve fitting: Prism, one site-specific binding with hill slope. Source data in the Source Data file.

Supplementary Figure 2

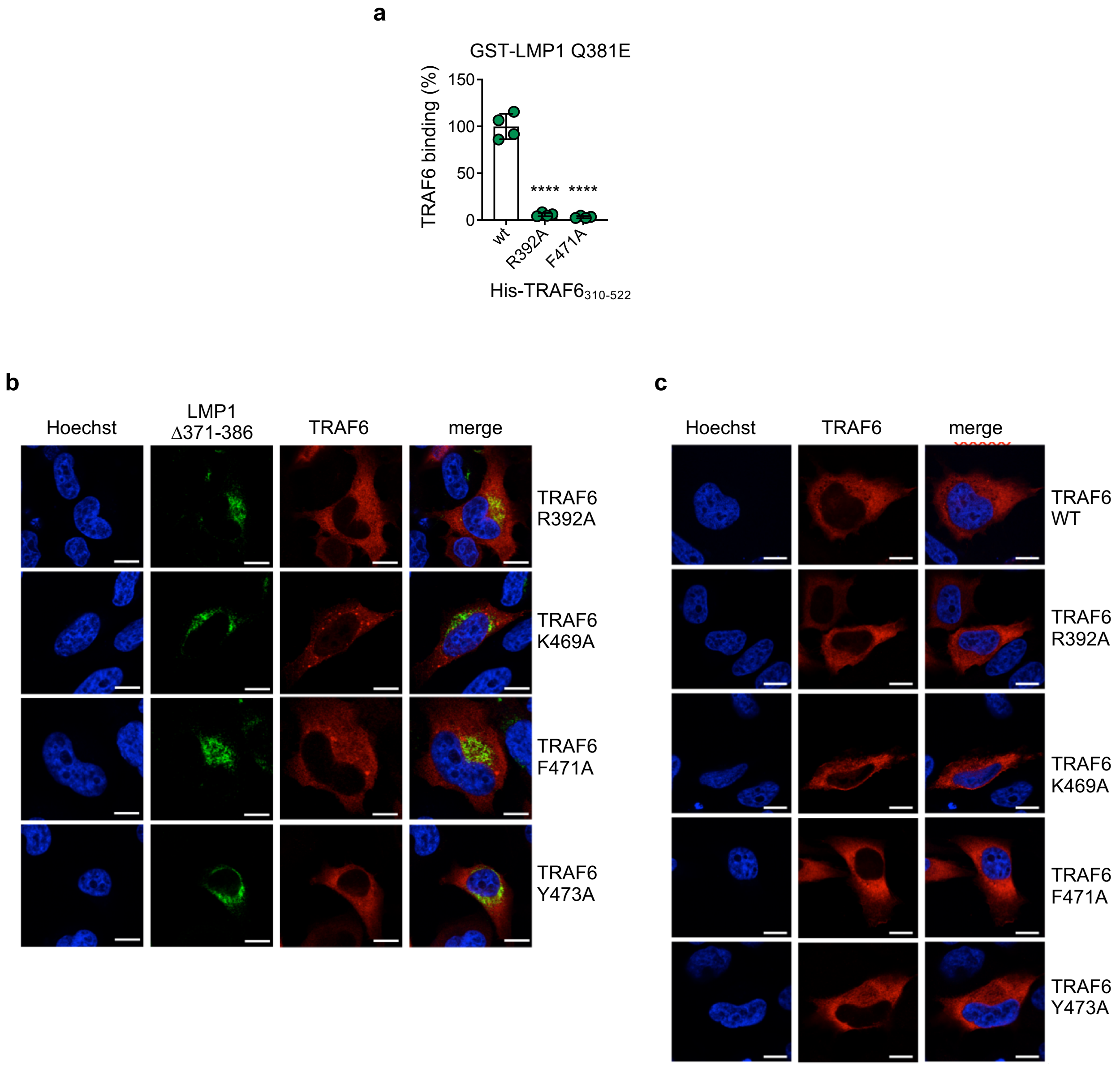

Supplementary Figure 2. LMP1 Q381E interaction with TRAF6 and additional controls to Figure 2e.

(a) The $TRAF6_{310-522}$ mutants $R_{392}A$ and $F_{471}A$ fail to interact with LMP1 Q381E. AlphaScreen PPI experiments. Data are mean values ± SD of four independent experiments. Statistics: one-way ANOVA. p-values: ****p ≤ 0.0001. Source data in the Source Data file.

(b) TRAF6 interacts with the sixteen C-terminal amino acids of CTAR2, as demonstrated by confocal microscopy. Neither TRAF6 wild-type nor any of the TRAF6 mutants colocalized with the LMP1 deletion mutant Δ371-386 in HeLa cells. Scale bar: 10 µm.

(c) Distribution of TRAF6 and the indicated TRAF6 mutants in HeLa cells in the absence of LMP1. Scale bar: 10 µm.

Supplementary Figure 3

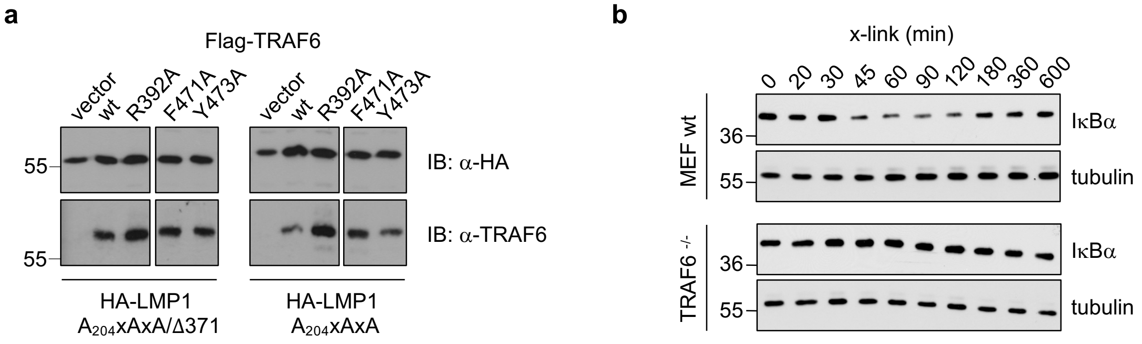

Supplementary Figure 3. Additional controls for the TRAF6 rescue experiments shown in Figure 3a.

(a) Expression levels of recombinant HA-LMP1 and Flag-TRAF6 proteins for the NF-κB rescue experiments shown in Figure 3a. Representative results.

(b) Absent canonical NF-κB activation by NGFR-LMP1 in TRAF6$^{-/-}$ MEFs, which had been transduced with NGFR-LMP1. NGFR-LMP1 activity was induced by antibody cross-linking for different times and IκBα levels were analyzed by immunoblotting. Tubulin served as the loading control. Representative blots are shown.

Supplementary Figure 4

a

TRAF6 - LMP1, RANK

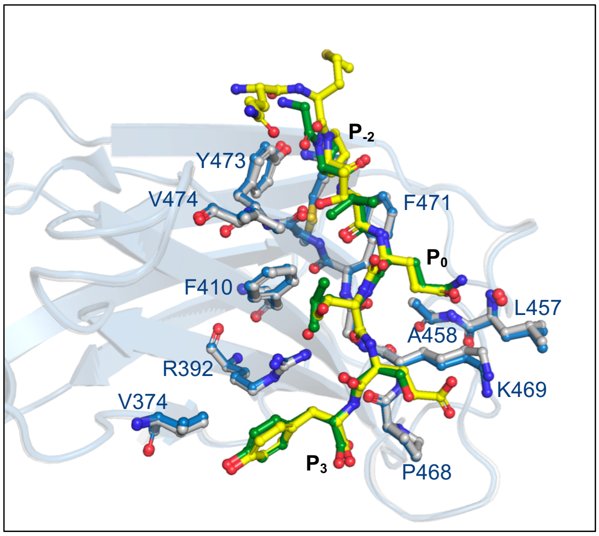

b

TRAF6 - LMP1, CD40

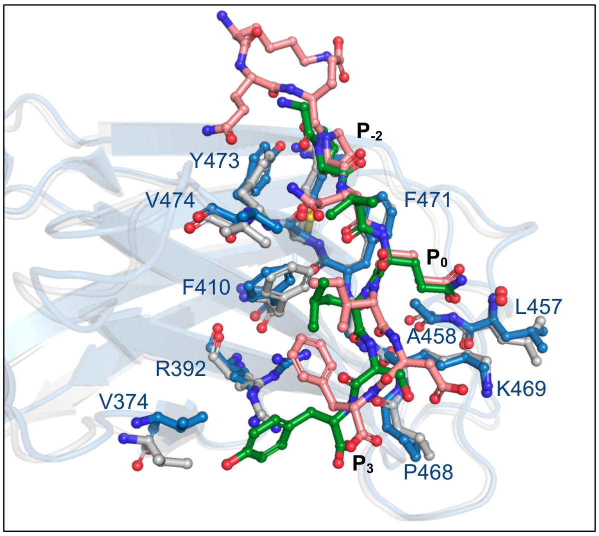

c

LMP1, RANK, CD40

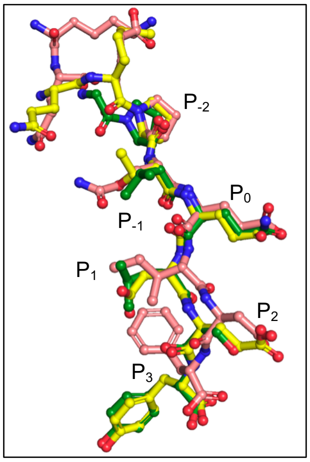

Supplementary Figure 4. Structure of an LMP1 Peptide compared to RANK and CD40 peptides on the surface of TRAF6.

(a) Overlay of the *in silico* model of LMP1 (green) and the crystal structure of RANK (PDB 1LB5, yellow) in complex with TRAF6 (blue with LMP1, gray with RANK).

(b) Overlay of the *in silico* model of LMP1 (green) and the crystal structure of CD40 (PDB 1LB6, salmon) in complex with TRAF6 (blue with LMP1, gray with CD40).

(c) Overlay of the LMP1 (green), RANK (yellow), and CD40 (salmon) peptides in complex with TRAF6.

Supplementary Figure 5

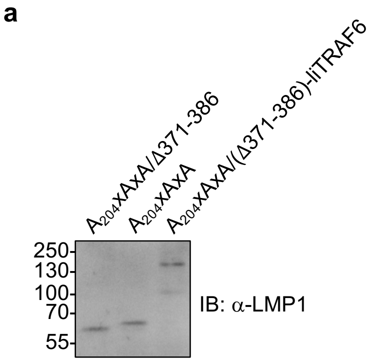

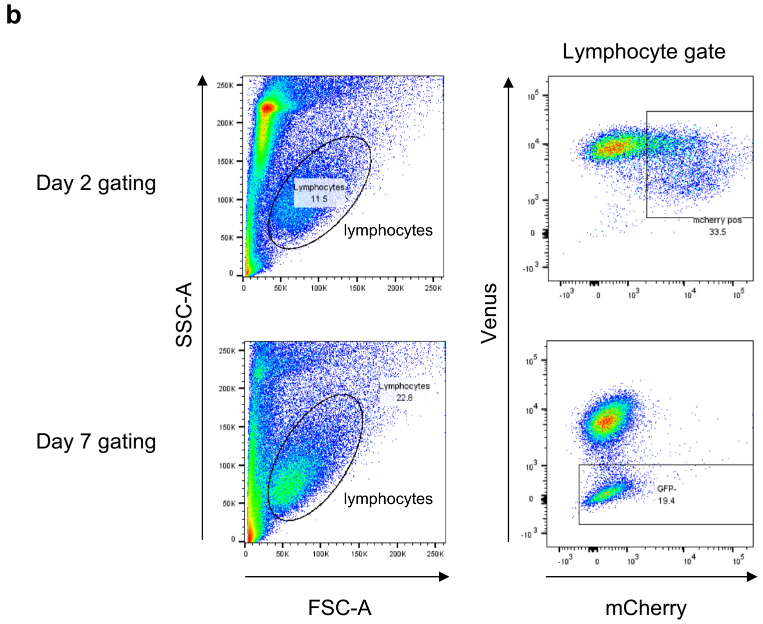

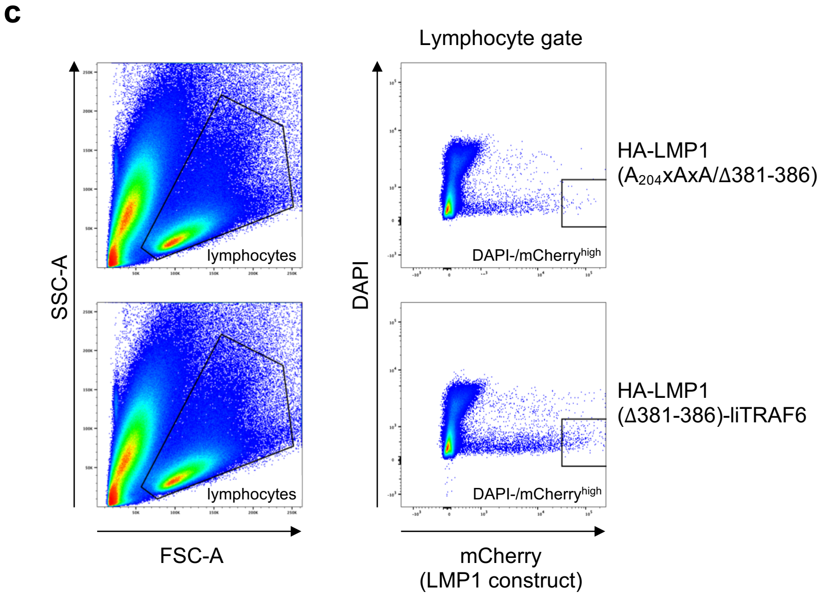

Supplementary Figure 5

Supplementary Figure 5. Protein expression of the LMP1-TRAF6 fusion protein and gating strategies.

(a) Expression levels of recombinant HA-LMP1 A_{204}xAxA/Δ371-386-liTRAF6 in MEF TRAF6$^{-/-}$. Representative results. Molecular weights are given in kilodaltons (kDa).

(b) Gating strategy for the flow cytometric analysis shown in Figures 6b and 6c.

(c) Gating strategy for the flow cytometric analysis shown in Figure 7c.

Supplementary Figure 6

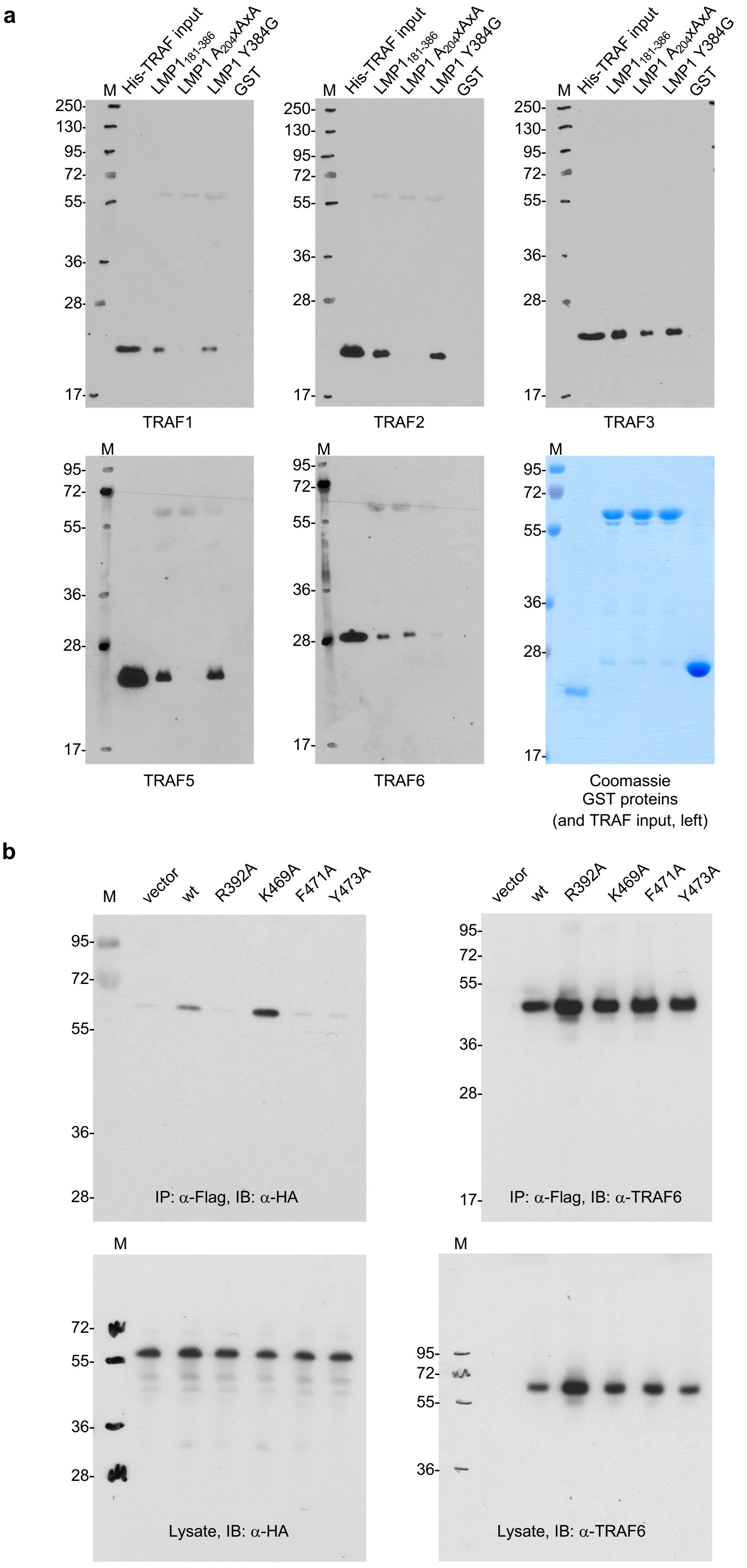

Supplementary Figure 6

c

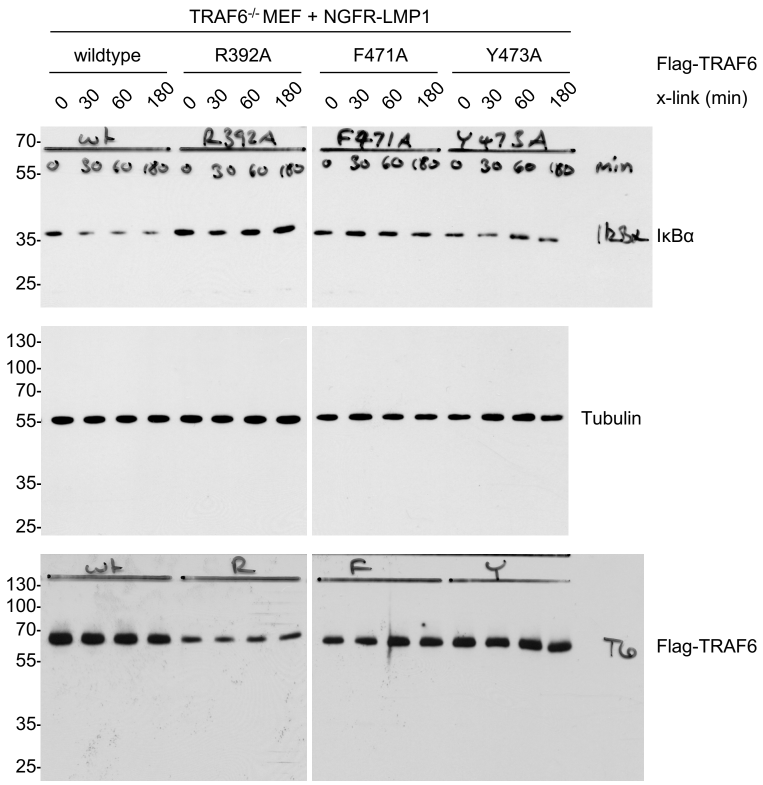

Supplementary Figure 6. Uncropped images of immunoblots

(a) Uncropped images of immunoblots shown in Figure 1b.

(b) Uncropped images of immunoblots shown in Figure 2d.

(c) Uncropped images of immunoblots shown in Figure 3c.

Molecluar weights are given in kilodaltons (kDa). M, molecular weight marker.
