# Supplementary material for: Epstein-Barr virus-driven B cell lymphoma mediated by a direct LMP1-TRAF6 complex
Source: Nat Commun. 2024 Jan 10;15:414. doi: 10.1038/s41467-023-44455-w (PMC10776578; doi:10.1038/s41467-023-44455-w)
Supplement: Supplementary file 3 — Reporting Summary [file 41467_2023_44455_MOESM3_ESM.pdf]

nature portfolio

Corresponding author(s): Arnd Kieser

Last updated by author(s): Dec 8, 2023

Reporting Summary

Nature Portfolio wishes to improve the reproducibility of the work that we publish. This form provides structure for consistency and transparency in reporting. For further information on Nature Portfolio policies, see our Editorial Policies and the Editorial Policy Checklist.

Statistics

For all statistical analyses, confirm that the following items are present in the figure legend, table legend, main text, or Methods section.

n/a	Confirmed	
☐	☒	The exact sample size (n) for each experimental group/condition, given as a discrete number and unit of measurement
☐	☒	A statement on whether measurements were taken from distinct samples or whether the same sample was measured repeatedly
☐	☒	The statistical test(s) used AND whether they are one- or two-sided<br>*Only common tests should be described solely by name; describe more complex techniques in the Methods section.*
☒	☐	A description of all covariates tested
☒	☐	A description of any assumptions or corrections, such as tests of normality and adjustment for multiple comparisons
☐	☒	A full description of the statistical parameters including central tendency (e.g. means) or other basic estimates (e.g. regression coefficient) AND variation (e.g. standard deviation) or associated estimates of uncertainty (e.g. confidence intervals)
☐	☒	For null hypothesis testing, the test statistic (e.g. F, t, r) with confidence intervals, effect sizes, degrees of freedom and P value noted<br>*Give P values as exact values whenever suitable.*
☒	☐	For Bayesian analysis, information on the choice of priors and Markov chain Monte Carlo settings
☒	☐	For hierarchical and complex designs, identification of the appropriate level for tests and full reporting of outcomes
☒	☐	Estimates of effect sizes (e.g. Cohen's d, Pearson's r), indicating how they were calculated

Our web collection on statistics for biologists contains articles on many of the points above.

Software and code

Policy information about availability of computer code

Data collection	Flow cytometry: BD FACSDiva 8.0.1., NMR data collection: Bruker BioSpin Topspin 3.2., Structural Modeling: Schrödinger platform for drug discovery (release 2021-3) by Schrödinger, Inc.
Data analysis	Confocal microscopy data analysis: Leica LAS AF software v.2.7.3.9723. Flow cytometry data analysis: FlowJo v.10.8.1. NMR spectra processing: NMRPipe NMRDraw v.8.7., NMR data analysis: CCPN Analysis software v.2.4.1, Statistics and graphics: GraphPad Prism v.10.1.0. Structural data presentation: Schrödinger PyMol Molecular Graphics System version 2.5.2 and PyMol 2.4.1.

For manuscripts utilizing custom algorithms or software that are central to the research but not yet described in published literature, software must be made available to editors and reviewers. We strongly encourage code deposition in a community repository (e.g. GitHub). See the Nature Portfolio guidelines for submitting code & software for further information.

Data

Policy information about availability of data

All manuscripts must include a data availability statement. This statement should provide the following information, where applicable:

- Accession codes, unique identifiers, or web links for publicly available datasets
- A description of any restrictions on data availability
- For clinical datasets or third party data, please ensure that the statement adheres to our policy

Unique cell lines are available upon request. vDetailed molecular cloning strategies and a PDB file of the LMP1-TRAF6 model are available upon request. The

following PDB files were used for modeling and structural analysis and are available in public databases: 1LB5 and 1LB6. Uncropped images of immunoblots are provided in the Supplementary Information. Raw data, details to the statistical analyses and, when possible, exact p-values are provided in the Source Data file.

Research involving human participants, their data, or biological material

Policy information about studies with human participants or human data. See also policy information about sex, gender (identity/presentation), and sexual orientation and race, ethnicity and racism.

Reporting on sex and gender	No research involving human participants, their data, or biological material was included in this study.
Reporting on race, ethnicity, or other socially relevant groupings	No research involving human participants, their data, or biological material was included in this study.
Population characteristics	No research involving human participants, their data, or biological material was included in this study.
Recruitment	No research involving human participants, their data, or biological material was included in this study.
Ethics oversight	No research involving human participants, their data, or biological material was included in this study.

Note that full information on the approval of the study protocol must also be provided in the manuscript.

Field-specific reporting

Please select the one below that is the best fit for your research. If you are not sure, read the appropriate sections before making your selection.

☒ Life sciences ☐ Behavioural & social sciences ☐ Ecological, evolutionary & environmental sciences

For a reference copy of the document with all sections, see nature.com/documents/nr-reporting-summary-flat.pdf

Life sciences study design

All studies must disclose on these points even when the disclosure is negative.

Sample size	No sample size calculation was performed. Sample size was at least three (or more, up to ten, as indicated) samples, which was sufficient to reach statistical significance in AlphaScreen assays, immunoprecipitations and immunoblot analyses, immunoflourescence analyses, reporter assays, MTT assays, FACS analyses and transfections. Sample sizes were chosen according to experimental experience.
Data exclusions	In AlphaScreen PPI assays the data points representing the Hook effect (saturation of beads) were excluded from calculations of the Hill slope, see the Source Data file. This approach is also described in Methods (Statistics section). Otherwise, no data were excluded.
Replication	The number of independent experiments (replications) are given in the Figure legends for each experiment. With the exception of Figure 1c, which incorporates two independent experiments (membranes) with each peptide spotted in duplicate, each experiment was performed at least three times to guarantee reproducibility of all experiments. The results were reproduced in all replications.
Randomization	No allocation of samples into experimental groups was performed. No covariates needed to be taken into account.
Blinding	Blinding was not relevant to our animal study, because allocation of animals into experimental groups was not included. Blinding in other experiments was not relevant or applicable due to the experimental procedures of biochemical and cell culture experiments. All samples were treated equally within one experiment and were analysed in parallel after completion of the experiment.

Reporting for specific materials, systems and methods

We require information from authors about some types of materials, experimental systems and methods used in many studies. Here, indicate whether each material, system or method listed is relevant to your study. If you are not sure if a list item applies to your research, read the appropriate section before selecting a response.

Materials & experimental systems

n/a	Involved in the study
	☒ Antibodies
	☒ Eukaryotic cell lines
☒	☐ Palaeontology and archaeology
	☒ Animals and other organisms
☒	☐ Clinical data
☒	☐ Dual use research of concern
☒	☐ Plants

Methods

n/a	Involved in the study
☒	☐ ChIP-seq
	☒ Flow cytometry
☒	☐ MRI-based neuroimaging

Antibodies

Antibodies used	For immunoblots: Primary antibodies: α-6*His-tag (clone 4A4 or 2F12, source: antibody facility of the Helmholtz Center Munich, hybridoma supernatants were used at a 1:10 dilution), α-TRAF6 (H-274, #sc-7221, Santa Cruz Biotech., used at 1:1000), α-HA-tag (12CA5, #11583816001, Sigma-Aldrich, f.c. 0.4 µg/mL), α-IkBα (C-21, #sc-371, Santa Cruz Biotech., 1:500) and α-Tubulin (B-5-1-2, #sc-23948, Santa Cruz Biotech., 1:500). Secondary antibodies: Horseradish peroxidase-coupled α-mouse (#7076, Cell Signaling Technology, 1:5000), horseradish peroxidase-coupled α-rabbit (#7074, Cell Signaling Technology, 1:5000).<br>For immunoprecipitations: α-Flag 6F7 (6F7, #SAB4200071, Sigma-Aldrich).<br>For the detection of recombinant TRAF protein on peptide filter arrays: goat α-TRAF6 (C-20, #sc-6223, Santa Cruz Biotech., 1:1000), rabbit α-TRAF2 (C-20, #sc-876, Santa Cruz Biotech., 1:1000). Secondary antibodies: Horseradish peroxidase-coupled α-goat IgG (#305-035-003, Dianova, 1:500), Horseradish peroxidase-coupled α-rabbit IgG (Cell Signaling Technology, #7074, 1:5000).<br>For immunostaining: Primary antibodies: α-HA (1:1000, clone 16B12, BioLegend) and α-DYKDDDDK (1:1000, clone L5, BioLegend). Secondary antibodies: goat α-mouse Alexa 488 (1:500, cross-adsorbed, #A-11029, Invitrogen), goat α-rat Alexa 555 (1:500, cross-adsorbed, #A-21434, Invitrogen).<br>For NGFR-LMP1 crosslinking: α-NGFR primary antibody (clone HB8737, ATCC, f.c. 1 µg/mL). Secondary antibody: α-mouse IgG/IgM (#115-005-068, Dianova, f.c. 10 µg/mL).
Validation	Information regarding validation, specificity and references of the commercial primary antibodies used in this study are available on the websites, in catalogues or datatsheets of the suppliers. All antibodies are approved for the application and species used in this study. In particular and publications listed therein: α-TRAF6 (H-274, #sc-7221, Santa Cruz Biotech.): https://datasheets.scbt.com/sc-7221.pdf, α-HA-tag (12CA5, #11583816001, Sigma-Aldrich): https://www.sigmaaldrich.com/deepweb/assets/sigmaaldrich/product/documents/865/567/roaha.pdf, α-IkBα (C-21, #sc-371, Santa Cruz Biotech.): https://datasheets.scbt.com/sc-371.pdf, α-Tubulin (B-5-1-2, #sc-23948, Santa Cruz Biotech.): https://datasheets.scbt.com/sc-23948.pdf, α-TRAF6 (C-20, #sc-6223, Santa Cruz Biotech): https://datasheets.scbt.com/sc-6223.pdf, α-TRAF2 (C-20, #sc-876, Santa Cruz Biotech): https://datasheets.scbt.com/sc-876.pdf, α-HA (clone 16B12, BioLegend): www.biolegend.com/en-ie/products/anti-ha-11-epitope-tag-antibody-11071, and α-DYKDDDDK (clone L5, BioLegend): www.biolegend.com/en-ie/products/purified-anti-dykddddk-tag-antibody-4905.<br>The α-6*His-tag antibodies have been published for the detection of purified His-tagged protein in pulldown assays as performed in the present work (de Jong et al., Sci. Signal. 6, ra27 (2013)).<br>The α-NGFR primary antibody HB8737 has been approved and used for the activation of NGFR-LMP1 signaling in several previous studies (e.g. Voigt et al., Nat. Commun. 11, 685 (2020)).

Eukaryotic cell lines

Policy information about cell lines and Sex and Gender in Research

Cell line source(s)	HEK293 and HeLa cells were directly obtained from the German Collection of Microorganisms and Cell Cultures (www.dsmz.de).<br>TRAF6-/- cells have been described (Lomaga et al., Genes Dev. 13, 1015-1024 (1999)) and were directly obtained from Tak W. Mak.<br>HA-LCL3 (Schneider et al., PLoS Biol. 6, e8 (2008)) and LCL.NGFR-LMP1 cells (Voigt et al., Nat. Commun. 11, 685 (2020)) have been generated by our laboratory and were taken from laboratory stocks.<br>EBV-negative BL41 Burkitt lymphoma (Lenoir et al., IARC Sci. Publ. 309-318 (1985)) and EBV-positive LCL721 cells (Kavathas et al.,Proc. Natl. Acad. Sci. USA. 77, 4251-4255 (1980)) have been described and were taken from own laboratory stocks.
Authentication	Authenticated HEK293 (#ACC 305) and HeLa (#ACC 57) cells were directly obtained from the German Collection of Microorganisms and Cell Cultures (www.dsmz.de). These cells have been authenticated by the German Collection of Microorganisms and Cell Cultures by STR analysis according to the global standard ANSI/ATCC ASN-0002.1-2021 (2021) and by cytogenetics. The identity of TRAF6-/- MEFs was confirmed by immunoblotting for TRAF6. LCLs were confirmed by immunoblotting for LMP1 or NGFR-LMP1, respectively. BL41 cells were not specifically authenticated. However, BL41 cells showed typical Burkitt lymphoma cell morphology.
Mycoplasma contamination	The cell lines were not tested for mycoplasma contamination.
Commonly misidentified lines<br>(See ICLAC register)	No commonly misidentified lines were used in this study.

Animals and other research organisms

Policy information about studies involving animals; ARRIVE guidelines recommended for reporting animal research, and Sex and Gender in Research

Laboratory animals	Mus Musculus, C57BL/6, females, tumor cells were derived from Rag2KO;cgammaKO animals older than 8 weeks. Housing conditions: Mice were kept in groups in IVC cages with a 12 h dark-light cycle at 22 ± 2 °C and 55 ± 5 % relative humidity. They had access to food and water ad libitum, as well as to nest building material and shelter.
Wild animals	No wild animals were used in this study.
Reporting on sex	Sex was not considered for the study design. There are no primary data disaggregated for sex. There is no indication that the results apply to only one sex.
Field-collected samples	No field-collected animals or samples were used in this study.
Ethics oversight	Animal experiments and animal housing were approved by the authorities of the City of Berlin (Landesamt für Gesundheit und Soziales Berlin): reference numbers G0049/15, G0374/13, and G0135/11

Note that full information on the approval of the study protocol must also be provided in the manuscript.

Plants

Seed stocks	No plants were used in this study.
Novel plant genotypes	No plants were used in this study.
Authentication	No plants were used in this study.

Flow Cytometry

Plots

Confirm that:

☒ The axis labels state the marker and fluorochrome used (e.g. CD4-FITC).

☒ The axis scales are clearly visible. Include numbers along axes only for bottom left plot of group (a 'group' is an analysis of identical markers).

☒ All plots are contour plots with outliers or pseudocolor plots.

☒ A numerical value for number of cells or percentage (with statistics) is provided.

Methodology

Sample preparation	Transduced and electroporated murine tumor cells (Figure 6) and LCLs (Figure 7c) were resuspended in PBS supplemented with 1% FCS, 1mM EDTA and 0.05% sodium azide for flow cytometry analysis.
Instrument	Becton Dickinson Aria Fusion (Figure 6), Becton Dickinson LSRFortessa (Figure 7)
Software	Data collection: BD FACSDiva 8.0.1<br>Data analysis: FlowJo 10.8.1
Cell population abundance	No sorting steps were performed before analysis by flow cytometry.
Gating strategy	Life cells were included into the analysis, cell debris was excluded.

☒ Tick this box to confirm that a figure exemplifying the gating strategy is provided in the Supplementary Information.
